# Supplementary material for: Chromothripsis during telomere crisis is independent of NHEJ, and consistent with a replicative origin
Source: Genome Res. 2019 May;29(5):737–49. doi: 10.1101/gr.240705.118 (PMC6499312; doi:10.1101/gr.240705.118)
Supplement: Supplemental Material [file supp_gr.240705.118_Supplemental_file_1.zip › contigs/annotated_contigs/DB48/contig.2.DB48_length_519_mean_cov_3.75722543353.docx]

**DB48_length_519_mean_cov_3.75722543353**

```
AATGACTAATTAACAATACTGATATTTTTCCTTCTGTCCATTTCACAAATATAAATATTAGAAAGCATATTAATTAAATTAAAAGGCAC
 >chr8:2507309-2507549 + E=8e-133
CAAACAGAAATTGATTTTTTTAACTTGTGAAAATGTCTAGGCAAAGACTATGTACATCAGTCTTTTTATTCCTATTTTCAGAATGAAGA

TTCATTACTATTATGAGTTGAATTATCTCCCTCGTAAAAAGATAAGGTGAAATCCTAACCCT|TTT|TTTTGTATTTAGAGAGACAGGC
                                                                  >chr8:2509340-2509616
GCTGAGACCTTCCTGCCATCTTCAATCGATGGATACTTGCTGAGCAGCTGTTCTGAGACATTGTTCTGGGGGCTGGGGATAAATAGCAG
 + E=2e-154
AAGACAGCCAGAGCCTCTCTGCCCTGGCGTCTGATAGCAAAGGGCAGTAAGTGTTTGTGACAATTTCAGGTGTTAAAAATTCTACTGGT

AGAAAAGAGACCCGAAAAGGATTGGTTAGAGAAAGGGTCAACCAACAAGCTCAGGGCAGAGTAGGTAGAGACCTGT
```
